# Indizoline^1^

**DOI:** 10.1107/S1600536809036915

**Published:** 2009-09-19

**Authors:** Hoong-Kun Fun, Wisanu Maneerat, Surat Laphookhieo, Suchada Chantrapromma

**Affiliations:** a X-ray Crystallography Unit, School of Physics, Universiti Sains Malaysia, 11800 USM, Penang, Malaysia; b School of Science, Mae Fah Luang University, Tasud, Muang Chiang Rai 57100, Thailand; c Crystal Materials Research Unit, Department of Chemistry, Faculty of Science, Prince of Songkla University, Hat-Yai, Songkhla 90112, Thailand

## Abstract

The title compound [systematic name: 1-meth­oxy-2-(3-methyl­but-2-en­yl)-9*H*-carbazole-3-carbaldehyde], C_19_H_19_NO_2_, is a natural carbazole which was isolated from the twigs of *Clausena lansium*. The carbazole ring system is essentially planar with a mean deviation of 0.0068 (10) Å. The aldehyde substituent is approximately co-planar with the attached benzene ring with a torsion angle of −8.58 (14)°, whereas the meth­oxy group is rotated out of the benzene plane with a torsion angle of −82.17 (11)°. The dihedral angle between the mean planes of the 3-methyl-2-butenyl group and the carbazole ring is 88.06 (5)°. An inter­molecular N—H⋯O inter­action connects the mol­ecules into a chain along the *a* axis. The crystal is further consolidated by a C—H⋯O hydrogen bond and two π–π inter­actions with centroid–centroid distances of 3.6592 (6) and 3.7440 (6) Å.

## Related literature

For bond-length data, see Allen *et al.* (1987 ▶). For background to carbazoles and their biological activity, see: Adebajo *et al.* (2009 ▶); Ito *et al.* (1998 ▶); Kumar *et al.* (1995 ▶); Lin (1989 ▶); Ng *et al.* (2003 ▶); Yang *et al.* (1988 ▶). For a related structure, see: Fun *et al.* (2007 ▶). For the stability of the temperature controller used in the data collection, see: Cosier & Glazer (1986 ▶).
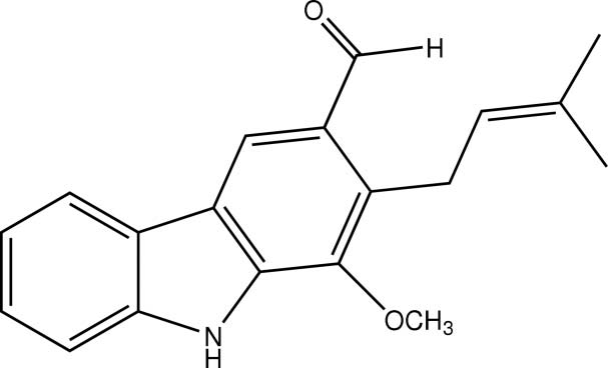


## Experimental

### 

#### Crystal data

C_19_H_19_NO_2_
*M* *_r_* = 293.35
Triclinic, 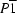

*a* = 9.0467 (1) Å
*b* = 9.3257 (1) Å
*c* = 10.6927 (1) Å
α = 65.717 (1)°
β = 86.994 (1)°
γ = 68.323 (1)°
*V* = 758.86 (2) Å^3^
*Z* = 2
Mo *K*α radiation
μ = 0.08 mm^−1^
*T* = 100 K
0.53 × 0.27 × 0.22 mm

#### Data collection

Bruker APEXII CCD area detector diffractometer
Absorption correction: multi-scan (**SADABS**; Bruker, 2005 ▶) *T*_min_ = 0.957, *T*_max_ = 0.982
20515 measured reflections
4410 independent reflections
3887 reflections with *I* > 2σ(*I*)
*R*_int_ = 0.023

#### Refinement

*R*[*F* ^2^ > 2σ(*F* ^2^)] = 0.042
*wR*(*F* ^2^) = 0.124
*S* = 1.05
4410 reflections
206 parameters
H atoms treated by a mixture of independent and constrained refinement
Δρ_max_ = 0.47 e Å^−3^
Δρ_min_ = −0.27 e Å^−3^

### 

Data collection: *APEX2* (Bruker, 2005 ▶); cell refinement: *SAINT* (Bruker, 2005 ▶); data reduction: *SAINT*; program(s) used to solve structure: *SHELXTL* (Sheldrick, 2008 ▶); program(s) used to refine structure: *SHELXTL*; molecular graphics: *SHELXTL*; software used to prepare material for publication: *SHELXTL* and *PLATON* (Spek, 2009 ▶).

## Supplementary Material

Crystal structure: contains datablocks global, I. DOI: 10.1107/S1600536809036915/is2459sup1.cif

Structure factors: contains datablocks I. DOI: 10.1107/S1600536809036915/is2459Isup2.hkl

Additional supplementary materials:  crystallographic information; 3D view; checkCIF report

## Figures and Tables

**Table 1:** Hydrogen-bond geometry (Å, °)

*D*—H⋯*A*	*D*—H	H⋯*A*	*D*⋯*A*	*D*—H⋯*A*
N1—H1*N*1⋯O2^i^	0.825 (19)	2.099 (19)	2.8843 (13)	158.8 (15)
C18—H18*A*⋯O1^ii^	0.96	2.59	3.5369 (15)	168

Symmetry codes: (i) 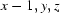; (ii) 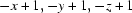.

**Fig. 1.**
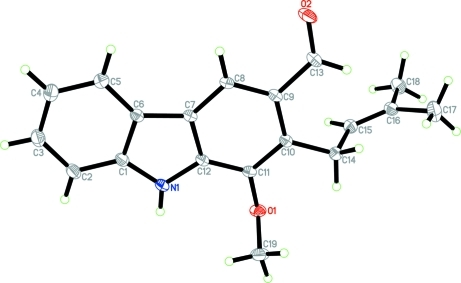
The structure of (I), showing 50% probability displacement ellipsoids and the atom-numbering scheme.

**Fig. 2.**
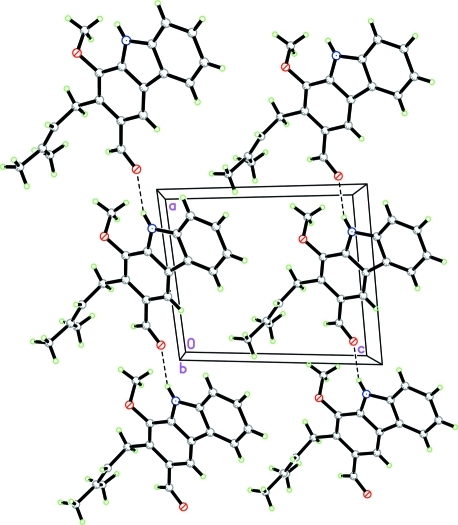
The crystal packing of (I) viewed along the b axis, showing one dimensional chains along the [1 0 0] direction. Hydrogen bonds are shown as dashed lines.

## supplementary crystallographic information

### Comment

*Clausena lansium* (Wampee) belongs to the Rutaceae family. Several parts of this plant have been used as folk medicine in China and Taiwan, for example, the leaves have been used for the treatment of coughs, asthma and gastro-intestinal diseases and the seeds for gastro-intestinal diseases such as acute and chronic gastro-intestinal inflammation and ulcers (Adebajo *et al.*, 2009). In addition, the fruits are used for influenza, colds and abdominal colic pains in Philippines (Lin, 1989). In previous studies, a number of coumarins (Ito *et al.*, 1998; Kumar *et al.*, 1995) and alkaloids (Lin, 1989; Yang *et al.*, 1988) have been isolated from different parts of this plant. As part of our continuing study on the chemical constituents and bioactive compounds from Thai medicinal plants, we report herein the crystal structure of the title compound (I), which was isolated from the twigs of *Clausena lansium* were collected from Nan province in the northern part of Thailand.

In the structure of (I), C_19_H_19_NO_2_ (Fig. 1), the carbazole ring system (C1–C12/N1) is essentially planar with a mean deviation of 0.0068 (10) Å. The aldehyde substituent is planarly attached to the benzene ring. The methoxy group is in an (+)-anti-clinal [torsion angle C19–O1–C11–C10 = 101.43 (10)°] whereas the 3-methyl-2-butenyl is in an (-)-*syn*-clinal [C9–C10–C14–C15 = -71.91 (11)°] conformation with respect to the attached benzene ring. The dihedral angle between the 3-methyl-2-butenyl moiety and the mean plane of carbazole ring is 88.06 (5)°. The bond lengths and angles in (I) are within normal ranges (Allen *et al.*, 1987) and are comparable to the related structure (Fun *et al.*, 2007).

In the crystal packing (Fig. 2), an N—H···O intermolecular interaction connects the molecules into one dimensional chains along the [1 0 0] direction. The crystal is further consolidated by C—H···O (Table 1) and π···π interactions with the *Cg*_1_···*Cg*_2_ distance = 3.7440 (6) Å and *Cg*_2_···*Cg*_3_ = 3.6592 (6) Å [symmetry code: (1 - *x*, 1 - *y*, -*z*) for both *Cg*···*Cg*]. *Cg*_1_, *Cg*_2_ and *Cg*_3_ are the centroids of C1–C6–C7–C12–N1, C1–C6 and C7–C12 rings, respectively.

### Experimental

Twigs of *Clausena lansium* (6.73 kg) were successively extracted with CH_2_Cl_2_ and acetone, over the period of 3 days each at room temperature to provide the crude CH_2_Cl_2_ and acetone extracts, respectively. The CH_2_Cl_2_ and acetone extracts were combined (34.02 g) and then subjected to quick column chromatography over silica gel eluted by gradient of hexane-acetone (100% hexane to 100% acetone) giving seventeen fractions (A—Q). Fraction G (207.1 mg) was subjected to purification by column chromatography using 20% EtOAc-hexane to yield the title compound (27.1 mg). Yellow block-shaped single crystals of the title compound suitable for *x*-ray structure determination were recrystallized from CH_2_Cl_2_/acetone (1:1, *v*/*v*) after several days (*m.p.* 344–345 K).

### Refinement

The H atom attached to N1 was located in a difference map and was isotropically refined. The remaining H atoms were placed in calculated positions with C—H = 0.93 Å for aromatic and CH, 0.97 for CH_2_ and 0.96 Å for CH_3_ atoms. The *U*_iso_ values were constrained to be 1.5*U*_eq_ of the carrier atom for methyl H atoms and 1.2*U*_eq_ for the remaining H atoms. A rotating group model was used for the methyl groups. The highest residual electron density peak is located at 0.70 Å from C8 and the deepest hole is located at 0.91 Å from C11.

### Crystal data

**Table:**

C_19_H_19_NO_2_	*Z* = 2
*M**_r_* = 293.35	*F*(000) = 312
Triclinic, *P*1	*D*_x_ = 1.284 Mg m^−^^3^
Hall symbol: -P 1	Melting point = 344–345 K
*a* = 9.0467 (1) Å	Mo *K*α radiation, λ = 0.71073 Å
*b* = 9.3257 (1) Å	Cell parameters from 4410 reflections
*c* = 10.6927 (1) Å	θ = 2.1–30.0°
α = 65.717 (1)°	µ = 0.08 mm^−^^1^
β = 86.994 (1)°	*T* = 100 K
γ = 68.323 (1)°	Block, yellow
*V* = 758.86 (2) Å^3^	0.53 × 0.27 × 0.22 mm

### Data collection

**Table:**

Bruker APEXII CCD area detector diffractometer	4410 independent reflections
Radiation source: sealed tube	3887 reflections with *I* > 2σ(*I*)
graphite	*R*_int_ = 0.023
φ and ω scans	θ_max_ = 30.0°, θ_min_ = 2.1°
Absorption correction: multi-scan (*SADABS*; Bruker, 2005)	*h* = −12→12
*T*_min_ = 0.957, *T*_max_ = 0.982	*k* = −13→13
20515 measured reflections	*l* = −15→15

### Refinement

**Table:**

Refinement on *F*^2^	Primary atom site location: structure-invariant direct methods
Least-squares matrix: full	Secondary atom site location: difference Fourier map
*R*[*F*^2^ > 2σ(*F*^2^)] = 0.042	Hydrogen site location: inferred from neighbouring sites
*wR*(*F*^2^) = 0.124	H atoms treated by a mixture of independent and constrained refinement
*S* = 1.05	*w* = 1/[σ^2^(*F*_o_^2^) + (0.0684*P*)^2^ + 0.2509*P*] where *P* = (*F*_o_^2^ + 2*F*_c_^2^)/3
4410 reflections	(Δ/σ)_max_ = 0.001
206 parameters	Δρ_max_ = 0.47 e Å^−^^3^
0 restraints	Δρ_min_ = −0.27 e Å^−^^3^

### Special details

**Table:**

Experimental. The crystal was placed in the cold stream of an Oxford Cryosystems Cobra open-flow nitrogen cryostat (Cosier & Glazer, 1986) operating at 100.0 (1) K.
Geometry. All e.s.d.'s (except the e.s.d. in the dihedral angle between two l.s. planes) are estimated using the full covariance matrix. The cell e.s.d.'s are taken into account individually in the estimation of e.s.d.'s in distances, angles and torsion angles; correlations between e.s.d.'s in cell parameters are only used when they are defined by crystal symmetry. An approximate (isotropic) treatment of cell e.s.d.'s is used for estimating e.s.d.'s involving l.s. planes.
Refinement. Refinement of *F*^2^ against ALL reflections. The weighted *R*-factor *wR* and goodness of fit *S* are based on *F*^2^, conventional *R*-factors *R* are based on *F*, with *F* set to zero for negative *F*^2^. The threshold expression of *F*^2^ > σ(*F*^2^) is used only for calculating *R*-factors(gt) *etc*. and is not relevant to the choice of reflections for refinement. *R*-factors based on *F*^2^ are statistically about twice as large as those based on *F*, and *R*- factors based on ALL data will be even larger.

### Fractional atomic coordinates and isotropic or equivalent isotropic displacement parameters (Å^2^)

**Table:**

	*x*	*y*	*z*	*U*_iso_*/*U*_eq_	
O1	0.26909 (8)	0.78512 (9)	0.28630 (7)	0.01884 (16)	
O2	0.90034 (9)	0.78623 (10)	0.07678 (8)	0.02297 (17)	
N1	0.23115 (10)	0.73264 (11)	0.03746 (9)	0.01646 (17)	
C1	0.26191 (11)	0.70558 (11)	−0.08151 (10)	0.01576 (18)	
C2	0.16804 (12)	0.67357 (12)	−0.15826 (11)	0.0196 (2)	
H2A	0.0692	0.6690	−0.1336	0.024*	
C3	0.22843 (13)	0.64886 (13)	−0.27314 (11)	0.0212 (2)	
H3A	0.1685	0.6274	−0.3264	0.025*	
C4	0.37714 (13)	0.65536 (13)	−0.31088 (10)	0.0204 (2)	
H4A	0.4140	0.6386	−0.3886	0.024*	
C5	0.47039 (12)	0.68656 (12)	−0.23362 (10)	0.01765 (19)	
H5A	0.5694	0.6902	−0.2585	0.021*	
C6	0.41224 (11)	0.71237 (11)	−0.11773 (9)	0.01449 (17)	
C7	0.47446 (11)	0.74608 (11)	−0.01497 (9)	0.01365 (17)	
C8	0.61476 (11)	0.76411 (11)	0.00817 (9)	0.01443 (17)	
H8A	0.6938	0.7538	−0.0505	0.017*	
C9	0.63649 (11)	0.79781 (11)	0.11998 (9)	0.01434 (17)	
C10	0.51798 (11)	0.81249 (11)	0.21241 (9)	0.01415 (17)	
C11	0.38006 (11)	0.78850 (11)	0.19182 (9)	0.01450 (18)	
C12	0.35762 (11)	0.75789 (11)	0.07777 (9)	0.01417 (17)	
C13	0.78581 (11)	0.81675 (12)	0.14115 (10)	0.01751 (19)	
H13A	0.7940	0.8543	0.2077	0.021*	
C14	0.54154 (12)	0.84670 (12)	0.33525 (10)	0.01731 (18)	
H14A	0.5751	0.9421	0.3046	0.021*	
H14B	0.4400	0.8784	0.3718	0.021*	
C15	0.66415 (12)	0.69530 (12)	0.44873 (10)	0.01723 (19)	
H15A	0.6619	0.5895	0.4679	0.021*	
C16	0.77577 (12)	0.69655 (12)	0.52484 (10)	0.01787 (19)	
C17	0.79953 (15)	0.85421 (14)	0.50890 (13)	0.0288 (2)	
H17A	0.7349	0.9490	0.4273	0.043*	
H17B	0.9103	0.8374	0.5010	0.043*	
H17C	0.7686	0.8769	0.5882	0.043*	
C18	0.88710 (13)	0.53395 (13)	0.63786 (11)	0.0229 (2)	
H18A	0.8557	0.4422	0.6485	0.034*	
H18B	0.8819	0.5463	0.7230	0.034*	
H18C	0.9947	0.5097	0.6141	0.034*	
C19	0.13064 (13)	0.94117 (15)	0.24419 (12)	0.0251 (2)	
H19A	0.0625	0.9333	0.3166	0.038*	
H19B	0.0730	0.9607	0.1621	0.038*	
H19C	0.1639	1.0339	0.2256	0.038*	
H1N1	0.145 (2)	0.7418 (19)	0.0698 (16)	0.029 (4)*	

### Atomic displacement parameters (Å^2^)

**Table:**

	*U*^11^	*U*^22^	*U*^33^	*U*^12^	*U*^13^	*U*^23^
O1	0.0144 (3)	0.0228 (3)	0.0189 (3)	−0.0072 (3)	0.0064 (3)	−0.0089 (3)
O2	0.0127 (3)	0.0318 (4)	0.0239 (4)	−0.0103 (3)	0.0028 (3)	−0.0097 (3)
N1	0.0107 (4)	0.0217 (4)	0.0199 (4)	−0.0077 (3)	0.0032 (3)	−0.0102 (3)
C1	0.0129 (4)	0.0159 (4)	0.0182 (4)	−0.0051 (3)	0.0004 (3)	−0.0070 (3)
C2	0.0148 (4)	0.0203 (4)	0.0247 (5)	−0.0065 (3)	−0.0012 (3)	−0.0103 (4)
C3	0.0208 (5)	0.0206 (4)	0.0226 (5)	−0.0067 (4)	−0.0033 (4)	−0.0100 (4)
C4	0.0237 (5)	0.0197 (4)	0.0182 (4)	−0.0073 (4)	0.0003 (4)	−0.0090 (3)
C5	0.0175 (4)	0.0177 (4)	0.0176 (4)	−0.0064 (3)	0.0026 (3)	−0.0077 (3)
C6	0.0123 (4)	0.0139 (4)	0.0161 (4)	−0.0043 (3)	0.0005 (3)	−0.0056 (3)
C7	0.0108 (4)	0.0138 (4)	0.0153 (4)	−0.0045 (3)	0.0014 (3)	−0.0052 (3)
C8	0.0105 (4)	0.0158 (4)	0.0160 (4)	−0.0050 (3)	0.0027 (3)	−0.0060 (3)
C9	0.0107 (4)	0.0147 (4)	0.0163 (4)	−0.0051 (3)	0.0006 (3)	−0.0050 (3)
C10	0.0120 (4)	0.0142 (4)	0.0153 (4)	−0.0047 (3)	0.0007 (3)	−0.0055 (3)
C11	0.0112 (4)	0.0160 (4)	0.0160 (4)	−0.0054 (3)	0.0034 (3)	−0.0064 (3)
C12	0.0102 (4)	0.0150 (4)	0.0166 (4)	−0.0048 (3)	0.0011 (3)	−0.0060 (3)
C13	0.0133 (4)	0.0201 (4)	0.0178 (4)	−0.0077 (3)	−0.0002 (3)	−0.0052 (3)
C14	0.0152 (4)	0.0192 (4)	0.0179 (4)	−0.0052 (3)	0.0007 (3)	−0.0093 (3)
C15	0.0171 (4)	0.0177 (4)	0.0166 (4)	−0.0071 (3)	0.0021 (3)	−0.0066 (3)
C16	0.0156 (4)	0.0200 (4)	0.0173 (4)	−0.0065 (3)	0.0017 (3)	−0.0075 (3)
C17	0.0303 (6)	0.0248 (5)	0.0321 (6)	−0.0115 (4)	−0.0063 (4)	−0.0109 (4)
C18	0.0216 (5)	0.0231 (5)	0.0200 (4)	−0.0078 (4)	−0.0023 (4)	−0.0053 (4)
C19	0.0161 (5)	0.0297 (5)	0.0280 (5)	−0.0041 (4)	0.0061 (4)	−0.0152 (4)

### Geometric parameters (Å, °)

**Table:**

O1—C11	1.3857 (11)	C9—C13	1.4658 (13)
O1—C19	1.4363 (13)	C10—C11	1.3895 (12)
O2—C13	1.2235 (12)	C10—C14	1.5155 (12)
N1—C12	1.3727 (11)	C11—C12	1.4018 (12)
N1—C1	1.3909 (12)	C13—H13A	0.9300
N1—H1N1	0.822 (17)	C14—C15	1.5071 (13)
C1—C2	1.3948 (13)	C14—H14A	0.9700
C1—C6	1.4113 (13)	C14—H14B	0.9700
C2—C3	1.3892 (14)	C15—C16	1.3360 (13)
C2—H2A	0.9300	C15—H15A	0.9300
C3—C4	1.3999 (15)	C16—C17	1.5035 (14)
C3—H3A	0.9300	C16—C18	1.5054 (14)
C4—C5	1.3895 (14)	C17—H17A	0.9600
C4—H4A	0.9300	C17—H17B	0.9600
C5—C6	1.3978 (13)	C17—H17C	0.9600
C5—H5A	0.9300	C18—H18A	0.9600
C6—C7	1.4500 (12)	C18—H18B	0.9600
C7—C8	1.3875 (12)	C18—H18C	0.9600
C7—C12	1.4167 (12)	C19—H19A	0.9600
C8—C9	1.3951 (13)	C19—H19B	0.9600
C8—H8A	0.9300	C19—H19C	0.9600
C9—C10	1.4271 (13)		
			
C11—O1—C19	113.68 (8)	N1—C12—C11	128.96 (9)
C12—N1—C1	108.62 (8)	N1—C12—C7	109.54 (8)
C12—N1—H1N1	128.2 (11)	C11—C12—C7	121.50 (8)
C1—N1—H1N1	122.8 (11)	O2—C13—C9	124.13 (9)
N1—C1—C2	128.87 (9)	O2—C13—H13A	117.9
N1—C1—C6	109.20 (8)	C9—C13—H13A	117.9
C2—C1—C6	121.92 (9)	C15—C14—C10	112.82 (8)
C3—C2—C1	117.16 (9)	C15—C14—H14A	109.0
C3—C2—H2A	121.4	C10—C14—H14A	109.0
C1—C2—H2A	121.4	C15—C14—H14B	109.0
C2—C3—C4	121.77 (9)	C10—C14—H14B	109.0
C2—C3—H3A	119.1	H14A—C14—H14B	107.8
C4—C3—H3A	119.1	C16—C15—C14	127.08 (9)
C5—C4—C3	120.78 (9)	C16—C15—H15A	116.5
C5—C4—H4A	119.6	C14—C15—H15A	116.5
C3—C4—H4A	119.6	C15—C16—C17	124.45 (9)
C4—C5—C6	118.63 (9)	C15—C16—C18	120.70 (9)
C4—C5—H5A	120.7	C17—C16—C18	114.84 (9)
C6—C5—H5A	120.7	C16—C17—H17A	109.5
C5—C6—C1	119.73 (9)	C16—C17—H17B	109.5
C5—C6—C7	133.90 (9)	H17A—C17—H17B	109.5
C1—C6—C7	106.36 (8)	C16—C17—H17C	109.5
C8—C7—C12	119.22 (8)	H17A—C17—H17C	109.5
C8—C7—C6	134.49 (8)	H17B—C17—H17C	109.5
C12—C7—C6	106.27 (8)	C16—C18—H18A	109.5
C7—C8—C9	119.55 (8)	C16—C18—H18B	109.5
C7—C8—H8A	120.2	H18A—C18—H18B	109.5
C9—C8—H8A	120.2	C16—C18—H18C	109.5
C8—C9—C10	121.40 (8)	H18A—C18—H18C	109.5
C8—C9—C13	118.14 (8)	H18B—C18—H18C	109.5
C10—C9—C13	120.46 (8)	O1—C19—H19A	109.5
C11—C10—C9	118.94 (8)	O1—C19—H19B	109.5
C11—C10—C14	119.26 (8)	H19A—C19—H19B	109.5
C9—C10—C14	121.76 (8)	O1—C19—H19C	109.5
O1—C11—C10	120.95 (8)	H19A—C19—H19C	109.5
O1—C11—C12	119.63 (8)	H19B—C19—H19C	109.5
C10—C11—C12	119.33 (8)		
			
C12—N1—C1—C2	179.57 (9)	C13—C9—C10—C14	−0.39 (13)
C12—N1—C1—C6	0.45 (10)	C19—O1—C11—C10	101.43 (10)
N1—C1—C2—C3	−179.21 (9)	C19—O1—C11—C12	−82.17 (11)
C6—C1—C2—C3	−0.19 (14)	C9—C10—C11—O1	173.69 (8)
C1—C2—C3—C4	0.09 (15)	C14—C10—C11—O1	−3.97 (13)
C2—C3—C4—C5	0.20 (15)	C9—C10—C11—C12	−2.73 (13)
C3—C4—C5—C6	−0.37 (14)	C14—C10—C11—C12	179.61 (8)
C4—C5—C6—C1	0.27 (14)	C1—N1—C12—C11	−179.49 (9)
C4—C5—C6—C7	179.54 (9)	C1—N1—C12—C7	−0.48 (10)
N1—C1—C6—C5	179.20 (8)	O1—C11—C12—N1	4.24 (15)
C2—C1—C6—C5	0.01 (14)	C10—C11—C12—N1	−179.30 (9)
N1—C1—C6—C7	−0.25 (10)	O1—C11—C12—C7	−174.67 (8)
C2—C1—C6—C7	−179.44 (8)	C10—C11—C12—C7	1.79 (14)
C5—C6—C7—C8	−0.63 (18)	C8—C7—C12—N1	−178.66 (8)
C1—C6—C7—C8	178.71 (10)	C6—C7—C12—N1	0.31 (10)
C5—C6—C7—C12	−179.38 (10)	C8—C7—C12—C11	0.44 (13)
C1—C6—C7—C12	−0.04 (10)	C6—C7—C12—C11	179.42 (8)
C12—C7—C8—C9	−1.64 (13)	C8—C9—C13—O2	−8.58 (14)
C6—C7—C8—C9	179.73 (9)	C10—C9—C13—O2	170.98 (9)
C7—C8—C9—C10	0.67 (13)	C11—C10—C14—C15	105.69 (10)
C7—C8—C9—C13	−179.77 (8)	C9—C10—C14—C15	−71.91 (11)
C8—C9—C10—C11	1.55 (13)	C10—C14—C15—C16	139.14 (10)
C13—C9—C10—C11	−177.99 (8)	C14—C15—C16—C17	−0.51 (17)
C8—C9—C10—C14	179.15 (8)	C14—C15—C16—C18	178.19 (9)

### Hydrogen-bond geometry (Å, °)

**Table:**

*D*—H···*A*	*D*—H	H···*A*	*D*···*A*	*D*—H···*A*
N1—H1N1···O2^i^	0.825 (19)	2.099 (19)	2.8843 (13)	158.8 (15)
C18—H18A···O1^ii^	0.96	2.59	3.5369 (15)	168

Symmetry codes: (i) *x*−1, *y*, *z*; (ii) −*x*+1, −*y*+1, −*z*+1.
